# Microsatellite genotyping of *Plasmodium vivax* infections and their relapses in pregnant and non-pregnant patients on the Thai-Myanmar border

**DOI:** 10.1186/1475-2875-12-275

**Published:** 2013-08-06

**Authors:** Supinya Thanapongpichat, Rose McGready, Christine Luxemburger, Nicholas PJ Day, Nicholas J White, Francois Nosten, Georges Snounou, Mallika Imwong

**Affiliations:** 1 Department of Clinical Tropical Medicine, Faculty of Tropical Medicine, Mahidol University, Bangkok, Thailand; 2 Shoklo Malaria Research Unit, Mae Sot, Tak Province, Thailand; 3 Mahidol Oxford Tropical Research Unit, Faculty of Tropical Medicine, Mahidol University, Bangkok, Thailand; 4 Centre for Vaccinology and Tropical Medicine, Churchill Hospital, Oxford, UK; 5 Sanofi Pasteur, Lyon, France; 6 INSERM UMR-S 945, F-75013, Paris, France; 7 Université Paris 6, Pierre & Marie Curie, Faculté de Médecine Pitié-Salpêtrière, Paris, France; 8 Department of Molecular Tropical Medicine and Genetics, Faculty of Tropical Medicine, Mahidol University, Bangkok, Thailand

**Keywords:** Genetic diversity, Malaria, Plasmodium vivax, Pregnancy, Relapse

## Abstract

**Background:**

*Plasmodium vivax* infections in pregnancy are associated with low birth weight and anaemia. This parasites species is also characterised by relapses, erythrocytic infections initiated by the activation of the dormant liver stages, the hypnozoites, to mature. Genotyping of *P. vivax* using microsatellite markers has opened the way to comparative investigations of parasite populations. The aim of the study was to assess whether there were any differences between the parasites found in pregnant and non-pregnant patients, and/or between the admission infections and recurrent episodes during follow-up.

**Methods:**

Blood samples were collected from 18 pregnant and 18 non-pregnant patients, who had at least two recurrent episodes during follow-up, that were recruited in two previous trials on the efficacy of chloroquine treatment of *P. vivax* infections on the Thai-Myanmar border. DNA was purified and the *P. vivax* populations genotyped with respect to eight polymorphic microsatellite markers. Analyses of the genetic diversity, multiplicity of infection (MOI), and a comparison of the genotypes in the samples from each patient were conducted.

**Results:**

The *P. vivax* parasites present in the samples exhibited high genetic diversity (6 to 15 distinct allelic variants found for the 8 loci). Similar expected heterozygosity (*H*_e_) values were obtained for isolates from pregnant (0.837) and non-pregnant patients (0.852). There were modest differences between the MOI values calculated for both admission and recurrence samples from the pregnant patients (2.00 and 2.05, respectively) and the equivalent samples from the non-pregnant patients (1.67 and 1.64, respectively). Furthermore, the mean number of distinct alleles enumerated in the admission samples from the pregnant (6.88) and non-pregnant (7.63) patients were significantly lower than that found in the corresponding recurrent episodes samples (9.25 and 9.63, respectively).

**Conclusions:**

The *P. vivax* populations circulating in inhabitants along the Thai-Myanmar border, an area of low malaria transmission, displayed high genetic diversity. A subtle increase in the multiplicity of *P. vivax* infections in pregnant patients suggests a higher susceptibility to infection. The higher allelic diversity in the relapse as compared to the admission samples in both patient groups is consistent with the hypothesis that a febrile episode promotes the activation of hypnozoites.

## Background

Malaria infection during pregnancy substantially increases the risk of morbidity and mortality to the mother, her foetus and the neonate, and thus constitutes an important public health problem [1]. In areas of high endemicity, the nefarious effects of infection by *Plasmodium falciparum* are most pronounced in primigravidae, and though less severe, infections in subsequent pregnancies remain associated with anaemia and low birth weight [2]. In areas of low endemicity, the relatively low levels of acquired immunity against malaria parasites increase susceptibility to more severe clinical falciparum episodes during pregnancies, which could result in foetal or maternal death [3]. The impact of infection by *Plasmodium vivax*, the most prevalent parasite in the Asia-Pacific region, is less pronounced than that associated with *P. falciparum*[4]. In a large-scale study conducted on the western border of Thailand, an area of low endemicity, *P. vivax* infections were more common in primigravidae than in multigravidae and were associated with mild maternal anaemia and increased risk of low birth weight [5]. Histopathological examination of the placentas from *P. vivax*-infected and treated women did not reveal any evidence of parasite sequestration or pathological changes [6], thus the pathogenic mechanisms remain unclear.

One salient biological characteristic of *P. vivax* is the formation of dormant liver stages, hypnozoites, by a proportion of the sporozoites inoculated by the infected mosquito. Hypnozoites remain uninucleate and metabolically quiescent for varying durations, that can extend to years, before resuming their development to form mature schizonts that then initiate a new erythrocytic episode upon merozoite release. In tropical areas, *P. vivax* infections have a short latent period (2 to 6 weeks), and the primary episode is often followed by a succession of relapses (every 3 to 4 weeks) that wane in frequency with time [7]. In the mid 1990’s, the recurrence rate for *P. vivax* infections treated with chloroquine and followed up for 63 days was found to be 63% (95% CI 57-69%) in non pregnant patients on the Thai-Myanmar border [8]. In a similar study conducted towards the end of the 2000’s in adults and children in the same region the recurrence rate following chloroquine treatment increased to 79.1% (95% CI, 73.5%–84.8%), probably because of increased prevalence of chloroquine resistant *P. vivax*[9]. Indeed, the first case of *P. vivax* high-grade resistance to chloroquine in pregnancy was reported recently from the same area [4]. The only drug available to eliminate hypnozoites, thus preventing relapses, is primaquine. Thus, in 1995–1996 when the first *P. vivax* recurrence in non pregnant patients on the Thai-Myanmar border was treated with primaquine and chloroquine the risk of having a further vivax episode within 2 months was reduced by 96% (95% CI 83-99%) [8]. However, primaquine is contraindicated in pregnancy and in 1986 to 1997 23% (149/634) of pregnant women had two or more parasitaemia episodes [5].

In recent years, reliable methods to genotype *P. vivax* populations using microsatellites have been developed [10,11]. Using this methodology it was revealed that *P. vivax* infections in patients from Thailand, Myanmar and India are often polyclonal, and that the genotype of the parasites in the first relapse following chloroquine treatment [12] differs from that of the initial admission *P. vivax* population in more than half of the patients. This suggests heterologous activation of hypnozoites, most probably acquired from earlier inoculations. This was supported by data from a study of relapses in infants and in their mothers pre- and post-partum [13] that showed that whereas admission and relapse infections are often genetically heterologous in the mothers, those observed in the children are generally homogeneous. In a recent study *P. vivax* genetic diversity was compared in pregnant and non pregnant patients in Colombia, and found to be similar in both groups [14]. The purpose of the study presented here is to expand knowledge on the genetic diversity of *P. vivax* in pregnancy beyond the few studies quoted above. Given the altered immunological and physiological status in pregnancy, it was considered important to ascertain whether the pattern of relapse and the genetic diversity of the *P. vivax* populations observed differed between pregnant and non-pregnant patients. To this end, a genetic analysis was conducted on archived samples that had been collected in the course of drug treatment studies conducted in villages along the Thai-Myanmar border.

## Methods

### Study site and collection of blood samples

The studies took place in the Shoklo Malaria Research Unit clinics on the Thailand-Myanmar border, an area of low (estimated entomologic inoculation rate of one or less infectious bites per year) and seasonal malaria transmission [15]. The samples were derived from two previous trials on the efficacy of chloroquine in the treatment of *P. vivax*. The group of *P. vivax*-infected non-pregnant women represented all those patients with two relapses recruited between July 1995 and July 1996 [8], and in pregnant women recruited between November 1998 and January 2000 [16]. In both patients groups *P. vivax* was confirmed by blood smear and given a treatment with chloroquine (Government Pharmaceutical Organization, Thailand) using the following schedule: 15 mg base/kg on the first day, followed by 5 mg base/kg daily on the second and third day (total 25 mg base/kg). The non-pregnant patients were followed up for 63 days and any recurrent parasitaemia was recorded, treated appropriately and the patient followed up for a further 63 days. Pregnant patients were followed up until delivery. At recurrence the treatment administered was the same as that on admission, except in non-pregnant patients where the second recurrence was treated with chloroquine combined with primaquine (0.25 mg/kg daily for 14 days). The study in non-pregnant women was approved by the Ethics Committee of Mahidol University and the Karen Refugee Committee [8], and that in pregnant women [16] by the Ethics Committee of the Faculty of Tropical Medicine of Mahidol University and the Ethics Committee of the London School of Hygiene & Tropical Medicine. Written informed consent was obtained from the patient for the publication of this report and any accompanying images.

### DNA extraction and microsatellite genotyping

The blood samples from the non-pregnant patients were collected and stored on filter paper (Whatman 3 MM), while those from the pregnant women were whole blood with EDTA as anticoagulant stored at −20°C. Genomic DNA was extracted from a punched out 13 mm diameter spot for the filter paper samples (equivalent to about 35 μl of whole blood) and from 200 μl of the EDTA whole blood, using the QiAamp Blood kit (Hilden, Germany) eluted in a final volume of 100 μl and stored at −20°C until use. The presence of *P. vivax* was confirmed in all the samples by nested PCR [17]. Eight extensively polymorphic microsatellite markers: Pv1.501, Pv3.27, Pv3.502, Pv6.34, Pv 8.504 [10] and MS1, MS5 and MS7 [11], were used to genotype the isolates using published protocols. Two microliters of the purified genomic DNA were used as a template for amplification, and in cases where a secondary amplification was carried out, it was initiated with 1 μl of the primary amplification product. The amplified fragments were analysed on an ABI 3130 Genetic Analyzer and by GeneMapper® software version 4.0 (Applied Biosystems) to measure the variable length in the samples.

### Data analysis

In any isolate the presence of one or more alleles at a particular locus was interpreted as a co-infection with two or more genetically distinct clones, i.e. multiple or polyclonal infections [10,18]. A locus was classed as having multiple alleles when the score of the minor peak was at least one-third the height of the predominant allele present for this locus. Samples from which the data was ambiguous were re-amplified (maximum peak height < 300 fluorescent units). The genetic diversity was measured using the predominated allele at each locus to calculate the expected heterozygosity (*H*_e_). The formula defined as *H*_e_ = 1/(1-n) (1-∑*pi*^2^) where *p* is the frequency of *i*^th^ allele. Expected heterozygosity (*H*_e_) ranges between 0 and 1, a value close to 1 indicated high genetic diversity levels in the population [19]. The multiplicity of infection (MOI) was also calculated as the maximum number of alleles observed at any locus. Multilocus linkage disequilibrium (LD) was calculated by using a standardized index of association (*I*^S^_A_) [20,21]. This test compares the variance (*VD*) of the number of alleles shared between all pairs of haplotypes observed in the population (*D*) with the variance expected under random association of alleles (*VE*) as follows: *I*^S^_A_ = (*VD*/*VE-*1) (*r*-1), where *r* is the number of loci analyzed [20]. *VE* is derived from 10,000 simulated data sets in which alleles were randomly reshuffled among haplotypes. Significant linkage disequilibrium is detected if *VD* is greater than 95% of the values derived from the reshuffled data sets. Data were analyzed with LIAN 3.1 [22]. Only the dominant alleles were considered to verify linkage. In order to detect possible bias due to equivocal assignment of haplotypes in multiple-clone infections, linkage disequilibrium (LD) was tested at three levels: (i) for all infections including those with more than one multi-allelic locus, (ii) for single clone infection, and (iii) for unique haplotypes only. In order to assess relatedness between isolates the genotype observed at reappearances with that one admission day were compared: two genotypes were classed as related if a genotype was either a mixture of 2 adjacent microsatellite alleles at any one locus of which one was present in the paired sample, or if alleles at one or two loci differed only by one tandem repeat [13].

## Results and discussion

A total of 116 *P. vivax* isolates were selected for inclusion in this study: 54 were derived from 18 non-pregnant patients who all had two recurrent episodes, and 62 were obtained from 18 pregnant patients who all had two recurrences, four of whom then had a third and two more a fourth recurrence. The characteristics of the patients are provided in Table 1. Non-pregnant patients tended to be of lower age and to have a more frequent history of *P. vivax* infections in the year preceding the date of recruitment to the study than the pregnant patients. They also tended to have higher temperature and parasitaemia on admission. As expected, pregnant patients had a lower haematocrit than non-pregnant patients. The mean interval time to the first reappearance did not significantly differ between the two groups, 45 days for the pregnant *vs* 43 days for the non-pregnant patients, nor did it differ for the second reappearance, 51 days *vs* 48 days. The second recurrence in non-pregnant women was treated with chloroquine and primaquine, and no further recurrences occurred. In the pregnant women the mean times to the third and fourth reappearances were 51 days and 32 days, respectively.

**Table 1:** **Characteristics of pregnant and non-pregnant *P. vivax *patients**

**Characteristics**	**Pregnant patients**	**Non-pregnant patients**	***p*-value**
	**N = 18**	**N = 18**	
Age (years)^a^	24 ± 7 (15–40)	14 ± 10 (5–46)	<0.001
Weight (kg)^a^	48 ± 6 (41–62)	29 ± 12 (13–48)	<0.001
**Previous *P. vivax *malaria**			
During previous (year)^b^	5/18 (27.8)	15/18 (83.3)	0.003
>1 episode during previous (year)^b^	1/5 (20.0)	8/15 (53.3)	0.436
Time since the last episode (days)^c^	62 (21–102)	65 (36–218)	0.432
**Previous *P. falciparum***			
During previous (year)^b^	4/18 (22.2)	7/18 (38.9)	0.469
>1 episode during previous year^b^	1/4 (25.0)	1/7 (14.3)	0.712
Time since the last episode (days)^c^	26 (21–98)	55 (44–362)	0.088
**Characteristics of infection at 1st genotype on day admission**	**N = 18**	**N = 18**	
Proportion with a history of fever^b^	13/18 (72.2)	18/18 (100.0)	0.205
Duration of fever (days)^c^	3 (1–7)	2 (1–7)	0.016
Proportion febrile^b^	6/18 (33.3)	10/18 (55.6)	0.314
Temperature (°C)^a^	37.1 ± 1.4 (35.5-39.7)	37.8 ± 1.0 (36.8-39.9)	0.134
Haematocrit (%)^a^	32 ± 6 (17–42)	37 ± 3 (31–42)	0.002
Parasitaemia (uL)^d^	943(32–11,492)	1,991(43–25,844)	0.248
Time to 1st reappearance (days)^c^	45 (25–71)	43 (35–52)	0.495
**Characteristics of patients on day of follow up**	**N = 44**	**N = 36**	
Proportion with a history of fever^b^	22/44 (50.0)	32/36 (88.9)	<0.001
Duration of fever in days (range)^e^	2 (1–3)	1 (1–3)	0.013
Proportion febrile^b^	11/44 (25.0)	29/36 (80.6)	<0.001
Mean temperature (°C)^a^	36.9 ± 1.1 (35.0-39.7)	38.1 ± 1.0 (35.8-40.8)	<0.001
Haematocrit (%)^a^	31 ± 3 (23–39)	38 ± 3 (30–46)	<0.001
Parasitaemia (uL)^d^	637 (16–3,624)	1,581 (36–22,272)	0.022
**Interval times to reappearance (days)**			
1st reappearance to 2nd reappearance (range)^c^	51 (28–98)	48 (33–64)	0.468
2nd reappearance to 3rd reappearance (range)^c^	51(28–66)	-	-
3rd reappearance to 4th reappearance (range)^c^	32(28–35)	-	-

^a^ mean ± SD (min-max).

^b^ Number of patients (%).

^c^ Median (min-max).

^d^ Geometric mean (min-max).

^e^ Range.

N = number of patient.

**Figure 1:**
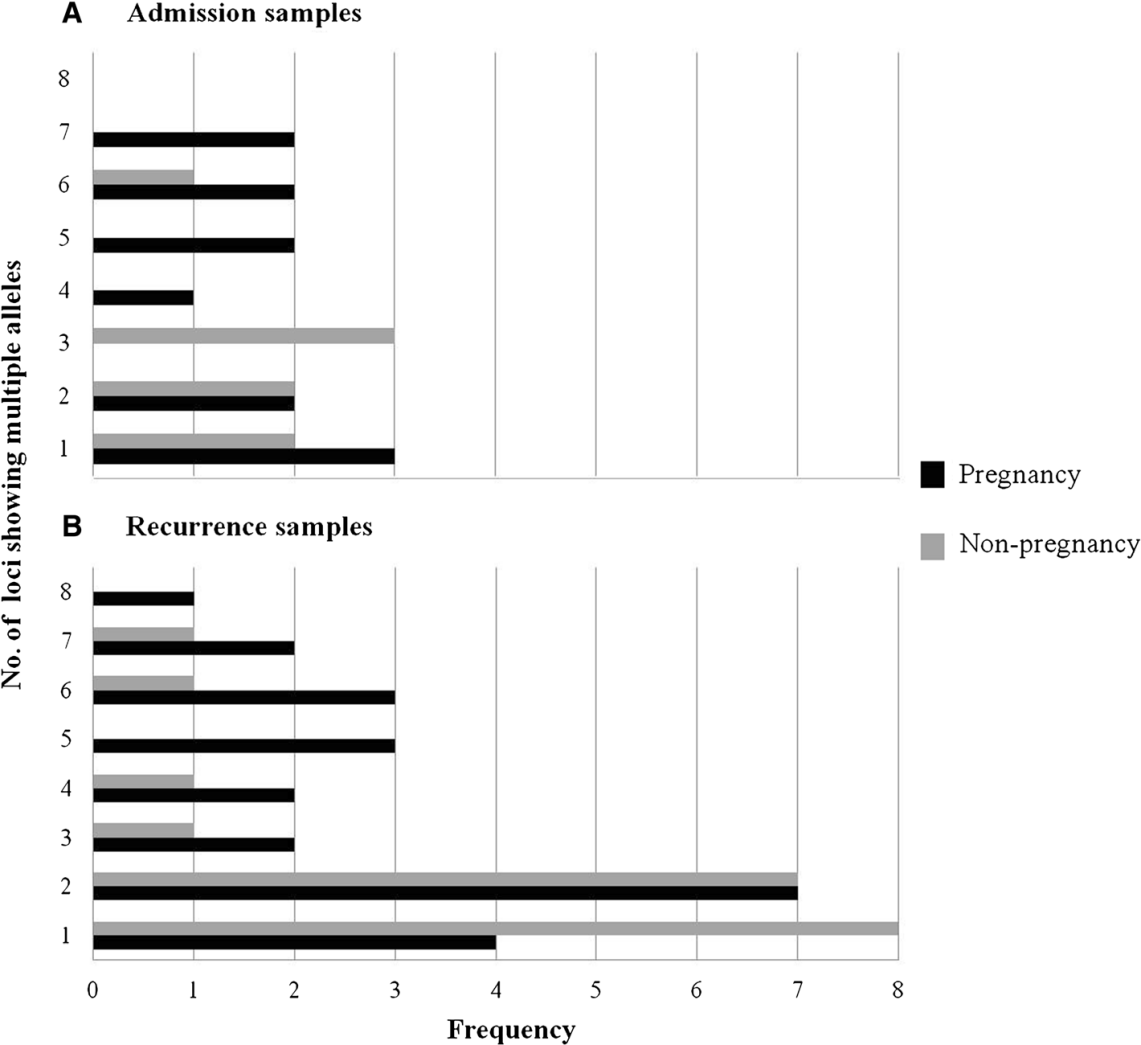
**Frequency distribution of the number of loci with multiple alleles.** The frequency of samples carrying multiple alleles for a given locus is plotted against the total number of loci in each sample found to be polyclonal.

The microsatellite genotyping data were used to calculate the mean number of distinct alleles (*A*), the heterozygosity (*H*_*e*_) and the mean number of distinct allelic variants for each locus in each sample (Table 2). The high number of distinguishable allelic forms observed for each locus and the high value of heterozygosity indicated that overall the *P. vivax* isolates circulating in the patients had a high degree of genetic diversity. The values did not significantly differ between parasites from pregnant and non-pregnant patients. There was a tendency that did not reach significance for samples from pregnant women to have a lower mean number of distinguishable alleles (*A*) than those from non-pregnant patients. On the other hand the number of distinct alleles per locus was significantly higher in pregnant *vs* non-pregnant patients. This was reflected in the higher MOI observed in the combined admission and recurrence samples from the pregnant *vs* non-pregnant women (Table 3), a difference that nearly reached significance. Nonetheless, the proportion of polyclonal infections was not statistically different between the samples obtained from either group (Table 3). When all the *P. vivax* samples from the admission *vs* the recurrent samples were compared (Table 2), there was a significant difference in the mean number of distinct alleles (A) observed for the loci (7.25 *vs* 9.5, p-value 0.0001), and the isolates from the recurrent episodes showed a higher proportion of loci for which more than one allelic variant was noted in each sample (Figure 1). Linkage disequilibrium was assessed for clonal infections, infections with unique haplotype and all infections, in samples from pregnant patients and non-pregnant patients that were subdivided as admission and recurrence samples (Table 4). No evident for linkage disequilibrium was found for the parasites in the samples obtained on admission, or in the subgroup of recurrence samples with a monoclonal infection. However, significant linkage disequilibrium was found for the recurrent samples from the two groups when considered in their entirety or for the subgroup that has a unique haplotype (Table 4).

**Table 2:** **Genetic diversity of *P. vivax *infections in pregnant and non-pregnant women based on 8 microsatellite markers**

**Episodes**	**Diversity**	**Patients**	**n**	***Plasmodium vivax *loci**								**Mean**	**SD**	**SE**	***p*-value**^***a***^
				**Pv 1.501**	**Pv 3.27**	**Pv 3.502**	**Pv 6.34**	**Pv 8.504**	**MS1**	**MS5**	**MS7**				
Admission samples	No. of distinct alleles (*A*)	P	18	9	9	7	7	6	5	6	6	6.88	1.46	0.52	0.265
		NP	18	8	12	9	8	6	4	9	5	7.63	2.56	0.91	
	*H*_*e*_	P	18	0.919	0.895	0.889	0.739	0.856	0.772	0.838	0.742	0.831	0.07	0.03	0.533
		NP	18	0.876	0.948	0.922	0.830	0.739	0.767	0.892	0.802	0.847	0.07	0.03	
	No. of distinct alleles per locus	P	18	1.59	1.61	1.50	1.22	1.56	1.35	1.27	1.38	1.44	0.14	0.05	<0.001
		NP	18	1.28	1.33	1.11	1.17	1.22	1.13	1.13	1.07	1.18	0.09	0.03	
Recurrent samples	No. of distinct alleles (*A*)	P	44	9	11	10	12	8	6	9	9	9.25	1.83	0.65	0.487
		NP	36	13	14	10	11	8	6	8	8	9.63	2.76	0.98	
	*H*_*e*_	P	44	0.882	0.824	0.872	0.901	0.846	0.736	0.848	0.839	0.844	0.05	0.02	0.316
		NP	36	0.911	0.919	0.884	0.867	0.808	0.783	0.874	0.835	0.86	0.05	0.02	
	No. of distinct alleles per locus	P	44	1.49	1.40	1.30	1.50	1.36	1.26	1.27	1.51	1.38	1.11	0.04	<0.001
		NP	36	1.26	1.31	1.11	1.17	1.11	1.06	1.10	1.23	1.17	0.09	0.03	
All samples	No of distinct alleles (*A*)	P	62	9	12	10	12	8	7	9	10	9.63	1.77	0.63	0.320
		NP	54	13	15	10	13	9	6	9	8	10.38	3.02	1.07	
	*H*_*e*_	P	62	0.885	0.846	0.869	0.864	0.845	0.736	0.834	0.820	0.837	0.05	0.02	0.340
		NP	54	0.906	0.921	0.888	0.848	0.781	0.770	0.866	0.836	0.852	0.06	0.02	
	No. of distinct alleles per locus	P	62	1.52	1.46	1.35	1.42	1.42	1.28	1.29	1.47	1.40	0.09	0.03	<0.001
		NP	54	1.26	1.31	1.11	1.17	1.15	1.08	1.11	1.18	1.17	0.08	0.03	

P = pregnant women and NP = non-pregnant women.

The No. of alleles *and H*_*e*_ values were calculated from a data set in which only the predominant alleles at each locus was considered.

The No. of alleles per locus values were calculated from all detected alleles at each loci, divided by the total number of samples.

^a^ Paired t-test was used to compare the means between groups.

**Table 3:** **Multiple clone infections and multiplicity of infection in *P. vivax***

**Episodes of infection**	**Pregnant women**	**Non-pregnant women**	***p*-value**	**Pregnant women**	**Non-pregnant women**	***p*-value**
	**Samples with multiple clones**			MOI*, mean (±SD)		
Admission	67%	44%	0.180	2.00	1.67	0.278
	(12/18 isolates)	(8/18 isolates)		(±0.97)	(±0.84)	
Recurrence	55%	53%	0.875	2.05	1.64	0.110
	(24/44 isolates)	(19/36 isolates)		(±1.41)	(±0.80)	
All episodes of infection	58%	50%	0.384	2.03	1.65	0.054
	(36/62 isolates)	(27/54 isolates)		(± 1.30)	(±0.81)	

*MOI = multiplicity of infection.

**Table 4:** **Linkage disequilibrium in *P. vivax***

			**Pregnant women**			**Non-pregnant women**	
		**n**	***I***_**A**_^**S**^	***p*-value**	**n**	***I***_**A**_^**S**^	***p*-value**
Single clone	Admission	6	0.0250	0.477	10	−0.0236	0.740
	Recurrence	20	0.1261	< 1.00 x 10^-04^	17	0.0141	0.257
Unique haplotype	Admission	18	0.0007	0.518	18	−0.0045	0.57
	Recurrence	40	0.0741	< 1.00 x 10^-04^	35	0.0323	1.00 x 10^-03^
All infections	Admission	18	0.0007	0.518	18	−0.0045	0.582
	Recurrence	44	0.0865	< 1.00 x 10^-04^	36	0.0385	1.00 x 10^-04^

**Figure 2:**
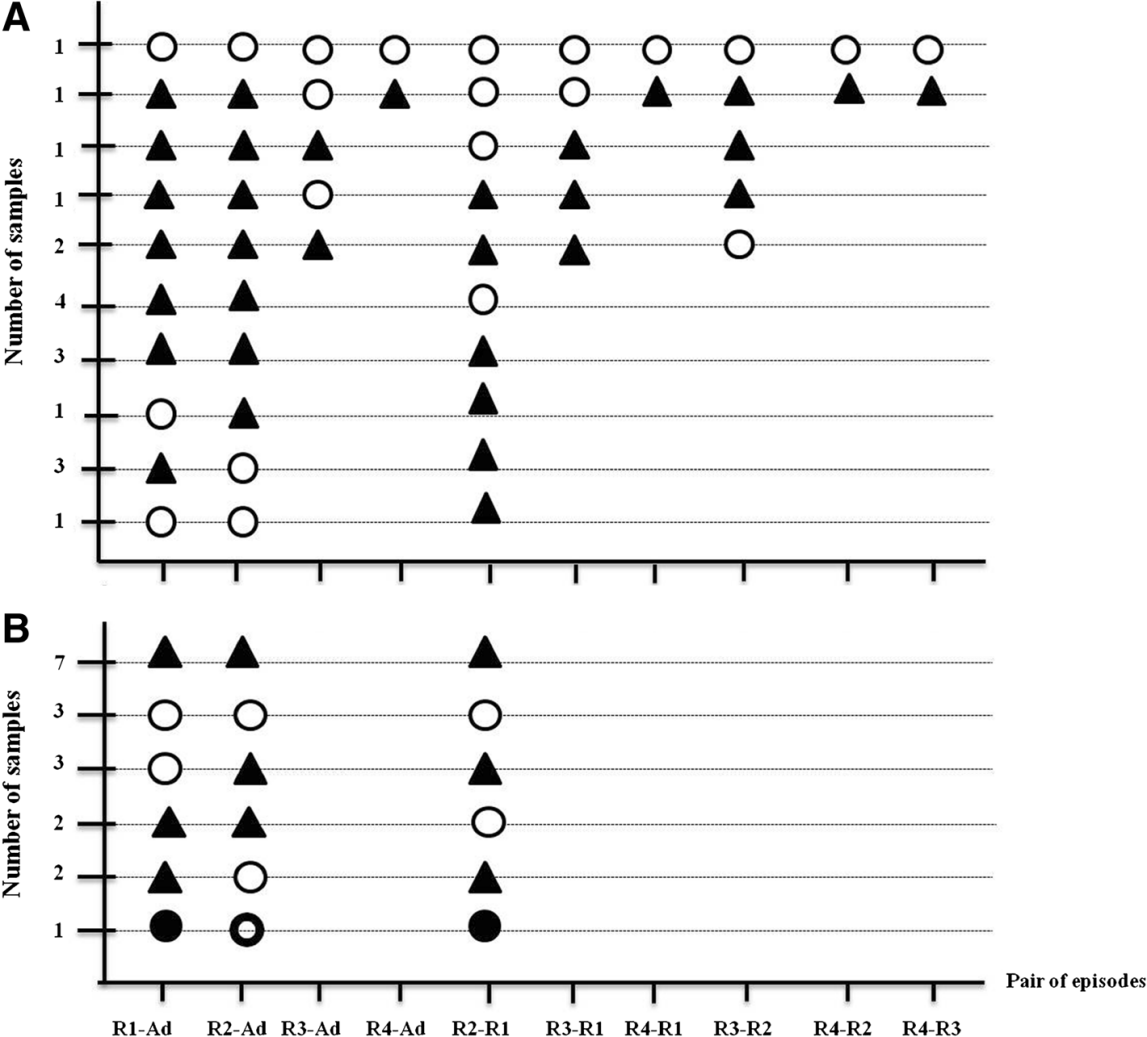
**Genotypic relatedness of the *P. vivax *isolates obtained from individual pregnant (A) and non-pregnant (B) patients.** The number of samples displaying each pattern is provided in the Y-axis. Pairwise comparisons were carried out for all possible combinations. The samples are coded as follows: Ad = admission, R1 = first recurrence, R2 = second recurrence, R3 = third recurrence, R4 = fourth recurrence. Two paired samples were classed as genetically different (▲) when the alleles variants differed by more than one repeat unit for at least one locus; the same if the alleles observed for all the loci are the same in the paired samples (○); and related if all or a subset of the allelic variants detected in one sample was also observed in the other paired sample. Given that two allelic variants from a given loci can differ by a single repeat unit because of artefactual slippage during amplification, related genotypes were classed as category “A” () if the allelic variants observed in a maximum of two loci differed by only by one repeat unit, and “B” (●) if three loci differed as above.

The genetic relatedness of the parasites obtained from each patient was assessed for all paired combinations (Figure 2, Table 5). Parasites from the first recurrent episode were different from those of the admission episode in 15 of the 18 pregnant patients and in 11 of the 18 non-pregnant patients, and a similar pattern was observed when the second recurrent episode parasites were compared to those of the first recurrence, in 11/18 and in 12/18 of the pregnant and non-pregnant patients, respectively. In only 1/18 and 3/18 patients from the two groups were all the episodes of the same genotype.

**Table 5:** **Comparison of the recurrent *P. vivax *genotypes**

	**Pregnant women**	**Non-pregnant women**	*p-*value*
	**N = 62**	**N = 54**	
No. of recurrent *Plasmodium vivax* infections	44	36	0.617
Median number of recurrences (range)	2 (2–4)	2 (2)	0.221
1st recurrence and admission episode similar	3/18	6/18	0.137
		1/18 B	
2nd recurrence and admission episode similar	5/18	5/18	0.167
		1/18 A	
3rd recurrence and admission episode similar	3/6	−	−
4th recurrence and admission episode similar	1/2	−	−
2nd and 1st recurrence episodes similar	7/18	5/18	0.729
		1/18 B	
3rd and 1st recurrence episodes similar	2/6	−	−
3rd and 2nd recurrence episodes similar	3/6	−	−
4th and 1st recurrence episodes similar	1/2	−	−
4th and 2nd recurrence episodes similar	1/2	−	−
4th and 3rd recurrence episodes similar	1/2	−	−
Proportion of genotypically similar recurrences	27/80	19/54	0.864
In genotypically different recurrence Median proportion of different alleles (%, rang)	62.50%	58.60%	0.562
	(range 12.5-100%)	(range 12.5-100%)	

Criteria for interpretation: A = 1 or 2 markers (loci) different with ≤1 possible stepwise mutation; B = 3 markers (loci) different but ≤1 repeat unit, different with ≤1 possible stepwise mutation.

* Chi-square test used to compare in two patients groups.

It is clear from the analyses above that the *P. vivax* populations in this hypoendemic region on the Thai-Myanmar border exhibited high genetic diversity, with about half of the isolates harbouring polyclonal infections. The differences between the parasites present in admission and recurrent samples, or those in pregnant and non-pregnant patients were relatively subtle. The complexity of the populations (as assessed by the mean number of distinct alleles for the 8 microsatellite markers) was higher in the recurrent as compared to the admission isolates in both groups of patients. The episodes in pregnant women tended to contain parasites with a slightly higher mean number of distinct alleles per locus and consequently a higher MOI. However, there are three caveats connected with sampling that need to be considered before drawing firm conclusions. First, given the relatively small differences, a higher number of patients might be needed. Second, the isolates were collected a few years apart. The fact that the parameters of genetic diversity did not significantly differ between the two groups of samples, suggests that there was little change in the extent of the parasites’ genetic diversity over the periods of sampling (1995–1999), though it is possible that the genetic diversity in the pregnant women does not reflect that in the general population. Third, the amount of blood equivalent to the DNA template aliquots used for genotyping was smaller for the non-pregnant (0.7 μl) than for the pregnant (4 μl) patients. This was inherent to the nature of the archived samples (dried blood spots and whole frozen blood, respectively). This six-fold difference in the volume of blood analyzed might account for the higher MOI in the pregnant women’s episodes, though it should be mitigated by the fact that mean parasitaemia in pregnant patients was half that in non-pregnant women, and that a 33% cut-off threshold was used to exclude the minor alleles. The main reason why this is unlikely is that it is inconsistent with the increase in allelic diversity in recurrent as compared to admission samples.

In conclusion, the parasites causing episodes in pregnant women had a higher genetic diversity than those in non-pregnant patients. This could be due to increased susceptibility to mosquito bites, reduced levels of immunity to infection, physiological changes in pregnancy especially if they affect reticulocyte dynamics, or a combination of these factors. In both patient groups the parasites at recurrence following treatment of the admission episode displayed a higher genetic complexity. In both pregnant and non-pregnant patients, the parasites at recurrence were genetically distinct from those on admission in about half the cases, as were the parasites from the first and the second recurrence. This pattern is similar to that observed earlier for relapsing *P. vivax* episodes in Thailand [12,13]. Differential accumulation of *P. vivax* in the placenta has not been noted in this area [6], though the phenomenon was observed in deliveries at Papua New Guinea [23], and *in vitro* cytoadhesion of *P. vivax*-infected red blood cells to ligands found in the placenta has been reported [24,25]. However, at the time when the samples analysed here were obtained, resistance of *P. vivax* to chloroquine, the treatment that was administered to both groups of patients, had not been recorded in Thailand [8,26]. Therefore, it is highly likely that the recurrent episodes analysed in this study were relapses originating from hypnozoites. The frequent heterologous nature of these relapse infections and their increased genetic diversity as compared to the admission infections are consistent with the activation of latent hypnozoites hypothesis [27].

## Competing interests

The authors declare that they have no competing interests.

## Authors’ contributions

RM, FN, MI, GS, ND and NW were involved in the conception and design of the study. ST performed the laboratory experiments and the analysis. RM, FN and CL were involved in the samples collection. ST and MI wrote the first draft. GS and MI wrote the final draft of the manuscript. All authors read and approved the final manuscript.
